# Proposing the “continuum of UTI” for a nuanced approach to antimicrobial stewardship

**DOI:** 10.1017/ash.2023.384

**Published:** 2023-09-29

**Authors:** Sonali Advani, Nicholas Turner, Rebecca North, Rebekah Moehring, Alicia Nelson, Valerie Vaughn, Charles Scales, Nazema Siddiqui, Kenneth Schmader, Deverick Anderson

## Abstract

**Background:** Historically, diagnosis of urinary tract infections (UTIs) has been divided into 3 categories based on symptoms and urine culture results: not UTI, asymptomatic bacteriuria (ASB), or UTI. However, some populations (eg, older adults, catheterized patients) may not present with signs or symptoms referrable to the urinary tract or have chronic lower urinary tract symptoms (LUTS), making the diagnosis of UTI challenging. We sought to understand the clinical presentation of patients who receive urine tests in a cohort of diverse hospitals. **Methods:** This retrospective descriptive cohort study included all adult noncatheterized inpatient and ED encounters with paired urinalysis and urine cultures (24 hours apart) from 5 community and academic hospitals in 3 states (NC, VA, GA) between January 1, 2017, and December 31, 2019. Trained abstractors collected clinical and demographic data using a 60-question REDCap survey. The study group met with multidisciplinary experts (ID, geriatrics, urology) to define the “continuum of UTI” (Table 1), which includes 2 new categories: (1) LUTS to capture patients with chronic lower urinary tract symptoms and (2) bacteriuria of unclear significance (BUS) to capture patients who do not clinically meet criteria for ASB or UTI (eg, older adults who present with delirium and bacteriuria). The newly defined categories were compared to current guideline-based categories. We further compared ASB, BUS, and UTI categories using a lower bacterial threshold of 1,000 colony-forming units. **Results:** In total, 220,531 encounters met study criteria. After using a random number generator and removing duplicates, 3,392 encounters were included. Based on current IDSA guidelines, the prevalence of ASB was 32.1% (n = 975), and prevalence of patients with “not UTI” was 1,614 (53%). Applying the expert panel’s new “continuum of UTI” definitions, the prevalence of “not UTI” patients decreased to 1,147 (37.7%), due to reassignment of 467 patients (15.3%)to LUTS. The prevalence of ASB decreased by 24% due to reassignment to BUS. Lowering the bacterial threshold had a slight impact on the number of definitive UTIs (14.9 vs 15.9%) (Table 1). **Conclusions:** Our rigorous review of laboratory and symptom data from a diverse population dataset revealed that diagnostic uncertainty exists when assessing patients with suspicion for UTI. We propose moving away from dichotomous approach of ASB versus UTI and using the “continuum of UTI” for stewardship conversations. This approach will allow us to develop nuanced deprescribing interventions for patients with LUTS or BUS (eg, watchful waiting, shorter course therapy) that account for the unique characteristics of these populations.

**Figure:**
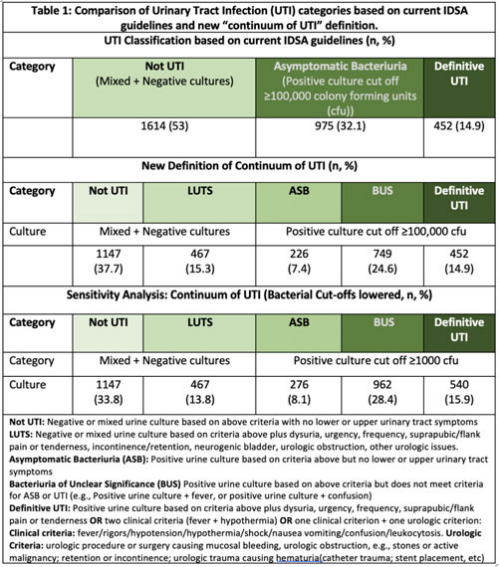

**Disclosures:** None

